# Long Non-Coding RNA CCAT2 Promotes the Development of Esophageal Squamous Cell Carcinoma by Inhibiting miR-200b to Upregulate the IGF2BP2/TK1 Axis

**DOI:** 10.3389/fonc.2021.680642

**Published:** 2021-07-27

**Authors:** Xiaodan Wu, Yihui Fan, Yupeng Liu, Biao Shen, Haimin Lu, Haitao Ma

**Affiliations:** ^1^Department of Thoracic Surgery, The First Affiliated Hospital of Soochow University, Suzhou, China; ^2^Department of Thoracic Surgery, Nantong Tumor Hospital, Nantong, China

**Keywords:** esophageal squamous cell carcinoma, long non-coding RNA CCAT2, microRNA-200b, insulin-like growth factor 2 mRNA-binding protein 2, competing endogenous RNA, thymidine kinase 1, N^6^-methyladenosine

## Abstract

Long non-coding RNAs (lncRNAs) have been shown to play important roles in human cancers, including esophageal squamous cell carcinoma (ESCC). In the current study, we identified CCAT2 as a relevant lncRNA and investigated its role in the progression of ESCC. RT-qPCR was adopted to detect CCAT2 expression in collected clinical samples, ESCC cell lines, and a normal cell line. We tested the correlation between CCAT2 expression and the prognosis of ESCC. RT-qPCR or immunoblotting was adopted to detect the expression of relevant factors in ESCC tissues or cells. Cell proliferation, apoptosis, migration, and invasion were examined by colony formation assay, flow cytometry, scratch assay, and Transwell assay, respectively, while subcutaneous tumorigenesis in nude mice was adopted to examine the role of CCAT2 in tumorigenesis of ESCC cells *in vivo*. Bioinformatics analysis, dual luciferase reporter assay, and RIP were conducted for the target relationship profiling. Me-RIP was adopted to detect m6A modification level of TK1 in ESCC tissues or cells. Upregulated CCAT2, IGF2BP2, and TK1 expression and inhibited miR-200b expression were observed in ESCC cells and tissues. CCAT2 bound to miR-200b and reduced its expression, leading to upregulated IGF2BP2 expression. IGF2BP2 improved TK1 mRNA stability to enhance its expression by recognizing its m6A modification. CCAT2 promoted the migration and invasion of ESCC cells *in vitro*, and tumorigenesis *in vivo* by upregulating TK1 expression, while overexpression of miR-200b reversed these effects of CCAT2. Overall, this study suggests that CCAT2 competitively binds to miR-200b to alleviate its inhibitory effects on IGF2BP2 expression, resulting in elevated TK1 expression, and an ensuing promotion of the development of ESCC.

## Introduction

Due to its increasing incidence esophageal cancer has become the 8^th^ most common cancer in the world and is the sixth leading cause of death among all cancer patients worldwide (1). Despite the best available treatments, esophageal cancer patients have a dismal outcome, with a five-year survival rate less than 10% (2). There are two main histological subtypes of esophageal cancer, namely esophageal squamous-cell carcinoma (ESCC) and esophageal adenocarcinoma (EAC) (3). The risk factors for these two subtypes differ, whereby causes of ESCC include smoking, excessive alcohol intake, and very hot drinks, while tobacco, obesity, and acid reflux are significant contributing factors for the development of EAC (4). Surgical excision supplemented with chemotherapy or chemoradiotherapy has been widely used as the standard treatment of advanced esophageal cancer, and in recent years immunotherapies are assuming an increasing role as alternative and effective treatment options for esophageal cancer patients diagnosed at different stages (5). Given the increasing incidence and poor prognosis of esophageal cancer, deciphering the regulating pathways of ESCC to facilitate the development of novel and efficient therapeutic strategies would be of great significance.

Long non-coding RNAs (lncRNAs) have been shown to play important roles in various human cancers, including ESCC (6). Colon cancer-associated transcript 2 gene (CCAT2) is significantly overexpressed in ESCC tissues, and more importantly, its expression is highly correlated with poor prognosis of ECSS patients (7). In addition, intensive study of microRNAs (miRs) has revealed their significant roles in the development pathways of multiple types of cancer (8). Interestingly, CCAT2 was reported to target miR-200b to regulate osteosarcoma cell progression (9). However, whether or how CCAT2 regulates miR-200b in the progression of ESCC remains unknown. Moreover, insulin-like growth factor 2 mRNA-binding protein 2 (IGF2BP2) is reported to be oncogene, which is highly related to ESCC (10), and furthermore the abnormal expression of thymidine kinase 1 (TK1) is regarded as an important clinical characteristic in patients with ESCC (11). As such, it seems plausible that both IGF2BP2 and TK1 are involved in the signaling axis initiated by CCAT2. Besides, IGF2BP2 was reported to be an m6A modification-related gene (12), suggesting that IGF2BP2 may tune TK1 expression through regulating the m6A modification of TK1 mRNA. In this study, we constructed cellular and animal models of ESCC to systematically investigate the signaling axis involving CCAT2.

## Materials and Methods

### Ethics Statement

All research procedures were conducted with approval of the Ethics Committee of Nantong Tumor Hospital (Nantong, Jiangsu) and in line with the *Declaration of Helsinki*. All patients and/or legal guardians signed the informed consent documentation prior to experiments. Animal experiments were approved by the Animal Ethics Committee of Nantong Tumor Hospital. Great efforts were made to minimize the number of animals used in the experiments and their suffering.

### Experimental Materials

Ninety-three patients (68 males and 25 females; aged 42-81 years, with a mean age of 62.12 ± 11.27 years) in seen the oncology department of Nantong Tumor Hospital from January 2015 to December 2018 were selected for surgical treatment, and pathologically confirmed as having ESCC after surgery. The patients had not received any treatment prior to the surgery. All surgical resection specimens were taken from the non-necrotic bleeding area in the center of the cancer tissue, and control samples consisted of the normal mucosa in the distal esophagus. The specimens were stored in the refrigerator at -80°C for later gene expression and histological analysis. ESCC cell lines (Eca109, TE-1, EC-1, and ESC410) and normal esophageal epithelial cell line HET-1A were purchased from American Type Culture Collection (ATCC; Manassas, VA, USA). Thirty-six SPF grade male BALB/c nude mice aged five weeks and weighing 18-22 g purchased from Shanghai SLAC Experimental Animal Co., Ltd. (Shanghai, China) were used for subcutaneous tumor xenografts experiments.

### Bioinformatics Analysis

Important lncRNAs and their downstream miRNA related to ESCC were identified through the previously reported literature. The RNA22 database was adopted to predict the binding site between lncRNA and miRNA and verify the targeting relationship. The downstream target genes of miRNA were predicted through miRWalk. Here, the |log_2_FoldChange (FC)| > 1.5 and *p* < 0.05 were used as thresholds to screen the differentially expressed genes of ESCC in the TCGA database to obtain the key downstream genes coding for relevant miRNAs. The binding site between miRNA and gene was predicted through RNA22, and their expression was obtained through GEPIA analysis. In brief, the limma package of R language was utilized to perform differential analysis on the ChIP-Seq datasets from GEO database, GSE20347, GSE29001, GSE38129, GSE45168, GSE45350, and GSE45670, with log2 transformation for GSE29001, GSE45168, GSE45350 and GSE45670 (**Supplementary Table 1**). Combined with GEPIA analysis, |log_2_FC| > 1 and *p* < 0.01 were used as thresholds to screen the differentially expressed genes of ESCC in TCGA database to obtain the intersection genes. The related genes of key genes were predicted through the online analysis websites Ualcan, LinkedOmics, GEPIA and MEM, and the intersect genes were combined to draw Venn diagrams, which identified six important key genes. The downstream pathways of these key genes were identified through the reported literature, and the expression correlation map and downstream gene expression trends were obtained from GEPIA analysis to further verify their regulatory relationship.

### Reverse Transcription Quantitative Polymerase Chain Reaction (RT-qPCR)

TRIzol reagent (Invitrogen, Calsbad, CA, USA) was adopted to extract total RNA from tissues and cells, and a NanoDrop 2000 micro-UV spectrophotometer (1011U, NanoDrop, Wilmington, DE, USA) was employed to detect the concentration and purity of the extracted total RNA. The RNA was reversely transcribed into complementary DNA (cDNA) as per the instructions of the TaqMan MicroRNA Assays Reverse Transcription primer (4427975, Applied Biosystems, NY, USA)/PrimeScript RT reagent Kit (RR047A, Takara, Tokyo, Japan). The primers for CCAT2, miR-200b, IGF2BP2, and TK1 were designed, and synthesized by TaKaRa (**Supplementary Table 2**). RT-qPCR was conducted on an ABI 7500 instrument (Applied Biosystems, Foster City, CA, USA), where the reaction conditions involved predenaturation at 95°C for 10 min, followed by 40 PCR cycles (denatured at 95°C for 10 s, annealed at 60°C for 20 s, and extended at 72°C for 34 s). The relative expression of mRNA or miRNA was normalized to glyceraldehyde-3-phosphate dehydrogenase (GAPDH) and calculated using the 2^-△△Ct^ method: △△Ct = △Ct_experimental group_ - △Ct_control group_, △Ct = Ct (target gene) - Ct (internal reference).

### Cell Culture and Grouping

Four ESCC cell lines Eca109, TE-1, EC-1, and ESC410, and the normal esophageal epithelial cell line HET-1A (all purchased from American Type Culture Collection, Manassas, VA, USA) were cultured with Roswell Park Memorial Institute (RPMI) 1640 medium containing 10% fetal bovine serum (FBS) in a 5% CO_2_ incubator at 37°C. After adherence to the wall, the cells were passaged and digested with 0.25% trypsin (Hyclone, Logan, UT, USA). The cells at the logarithmic growth phase were collected for subsequent experiments.

The cells were divided into the following groups: short hairpin RNA-negative control (sh-NC) group (transfected with interference control plasmid); sh-CCAT2 group (transfected with CCAT2 interference plasmid); overexpression (oe)-NC group (transfected with overexpression control plasmid); oe-CCAT2 group (transfected with CCAT2 overexpression plasmid); oe-NC + mimic NC group (transfected with overexpression control plasmid and miR-200b overexpression NC sequence); oe-CCAT2 + mimic NC group (transfected with CCAT2 overexpression plasmid and miR-200b overexpression NC sequence); oe-CCAT2 + miR-200b mimic group (transfected with CCAT2 overexpression plasmid and miR-200b overexpression sequence); oe-CCAT2 + sh-TK1 group (transfected with CCAT2 overexpression plasmid and TK1 interference plasmid). Transfected plasmids, mimic, and inhibitor were purchased and synthesized by Sino biological company (Beijing, China). The cells were seeded into six-well plates 24 h before transfection. Upon attaining 80% cell confluence, each plasmid was transiently transfected into ESCC cells as per the instructions of Lipofectamine 2000 transfection reagent (Invitrogen, Carlsbad, CA, USA). The medium was renewed after 6 h of transfection. After 48 h of culture, the cells were collected for subsequent experiments.

### Fluorescence In Situ Hybridization (FISH)

Subcellular localization of CCAT2 in cells was determined by FISH using the Ribo™ lncRNA FISH Probe Mix (Red) (Guangzhou RiboBio Co., Ltd., Guangzhou, Guangdong China). In brief, a cover glass was placed in a 6-well culture plate, where ESC410 cells were seeded at a density of 1 × 10^5^ cells/well. Upon reaching about 80% cell confluence, the cover glass was removed, washed with phosphate-buffered saline (PBS), and fixed at room temperature with 4% paraformaldehyde (1 mL). The cells were then treated with proteinase K (2 μg/mL), glycine, and acetamidine reagent, added with 250 μL prehybridization solution and incubated at 42°C for 1 h. The prehybridization solution was removed and the cells were added with 250 μL hybridization solution (RiboBio) containing the probe (300 ng/mL), and were then hybridized overnight at 42°C. After three rinses with phosphate-buffered saline-Tween-20 (PBST), 4’,6-diamidino-2-phenylindole (DAPI) (1: 800, diluted in PBST) dye solution was added to the plate to stain the nucleus for 5 min. Following PBST washing, the cells were mounted in fluorescence decay resistant medium. Five different visual fields were randomly selected under a fluorescence microscope (Olympus Optical Co., Ltd., Tokyo, Japan) for observation and photography.

### Colony Formation Assay

ESC410 cells at the logarithmic growth phase were digested with 0.25% trypsin and gently dispersed into single cells. The viable cells were counted and the cell density was adjusted to 1 × 10^6^ cells/mL. Cells were seeded into a dish containing 10 mL of 37°C preheated culture solution at a gradient density of 50, 100, and 200 cells/dish, and gently rotated to disperse the cells. The cells were cultured in a 37°C incubator under 5% CO_2_ for 2-3 weeks. When there were visible clones in the petri dish, the culture was concluded. The supernatant was discarded and the cells were carefully rinsed twice with PBS. The cells were fixed with 5 mL of 4% paraformaldehyde, and then the fixative was removed. The cells were stained using an appropriate amount of GIMSA (Invitrogen) for 10-30 min and dried in the air after the staining solution had been slowly washed off under running water. The stained dish was placed under an inverted microscope, and the number of cell clones was observed to calculate the rate of clone formation = number of clones formed/number of cells seeded.

### Flow Cytometry

Annexin V-fluorescein isothiocyanate (FITC)/propidium iodide (PI) double staining was adopted to detect cell apoptosis. ESC410 cells were seeded into 6-well plates at a density of 2 × 10^5^ cells/well. The cells in the blank group, NC group, and cell transfection group were transfected at a concentration of 100 nM. After 72 h, the culture medium was discarded and the adherent cells digested with trypsin. The cells were collected into a 15 mL centrifuge tube and the supernatant was discarded after centrifugation at 800 g. The cells were resuspended in 500 μL of binding buffer as per the instructions of the Annexin V-FITC Apoptosis Detection Kit Ι (Becton Dickinson and Company [BD], Franklin Lakes, New Jersey, USA). The cells were added with 5 μL FITC and 5 μL PI in the dark, mixed thoroughly, and incubated for 15 min. Cell apoptosis was detected by a flow cytometer (FACSCalibur; BD, San Jose, CA, USA).

### Transwell Assay

ESC410 cells were digested after starvation in serum-free medium for 24 h, and resuspended with serum-free medium Opti-MEMI (Invitrogen) containing 10 g/L bovine serum albumin (BSA, Sigma-Aldrich Chemical Company, St Louis, MO, USA) to a density of 3 × 10^4^ cells/mL. A Transwell chamber (24-well insert; pore size, 8 μm; Corning Incorporated, Corning, NY, USA) was employed for cell migration and invasion assays. Three chambers were set for each group, whereby 100 μL of cell suspension was added into each chamber. The 600 μL of 10% RPMI 1640 medium was added dropwise to the lower chambers, and incubated at 37°C under 5% CO_2_. In the invasion experiment, 50 μL Matrigel (Sigma-Aldrich) was placed in the chamber prior to the experiment, and after 48 h in culture, the cells were fixed and stained as mentioned above. The number of migrating stained cells was counted under an inverted microscope. Five random fields of view were selected for counting, and the average value was taken to evaluate the migration and invasion ability of cells.

### Scratch Assay

After 48 h of transfection, ESC410 cells were seeded into 6-well plates at a density of 5 × 10^5^ cells/well. After the cells had completely adhered to the wall of plates, a 2 mm cell scraper was adopted to scratch in the middle of each well, and the cells were cultured for a further 24 h. Photographs were taken at 0 h and 24 h after the scratches, respectively, and the healing ratio was calculated using image-Pro plus 6.0.

### Immunoblotting

ESCC cells were lysed with RIPA lysis buffer (Beyotime Biotechnology, Shanghai, China) on ice for 5 min, centrifuged at 14,000 rpm at 4°C, and the supernatant was collected. The protein concentration was determined using a bicinchoninic acid (BCA) kit (Pierce, Thermo Fisher, Austin, Texas, USA). Proteins were separated by 4 or 10% sodium dodecyl sulfate-polyacrylamide gel electrophoresis (SDS-PAGE) and then transferred to a polyvinylidene fluoride (PVDF) membrane. The membrane was blocked with 5% skimmed milk at room temperature for 1 h, and probed at 4°C overnight with diluted primary antibodies: anti-rabbit IGF2BP2 (ab173053, 1: 2000-1: 5000; Abcam, Cambridge, UK) and anti-rabbit TK1 (ab128431, 1: 5000-1: 50000; Abcam). The next day, the membrane was re-probed with diluted horseradish peroxidase (HRP)-labeled goat anti-rabbit IgG (Santa Cruz, CA, USA) at room temperature for 1 h. The immunoblots were visualized using enhanced chemiluminescence (EMD Millipore, Billerica, MA, USA) reagent under the Bio-Rad ChemiDoc ™ imaging system. Image J software (Bio-Rad, Hercules, CA, USA) was adopted to quantify the gray value of target protein with GAPDH as internal reference.

### Dual Luciferase Reporter Assay

The predicted binding site fragments and mutated fragments of CCAT2 and miR-200b were inserted into the luciferase reporter vector as reporter plasmids, CCAT2-wild type (WT) and CCAT2-mutant (MUT). CCAT2 luciferase reporter plasmid was co-transfected with NC or miR-200b mimic, respectively, into the 293T cells (Oulu Biotechnology, Guangzhou, China) to test whether CCAT2 could bind to miR-200b, where Renilla luciferase served as internal reference. After 48 h of transfection, the cells were collected and lysed. The luciferase detection kit (K801-200, Biovision, San Francisco, USA) was adopted, and the luciferase reporter gene detection was performed using a dual luciferase reporter gene analysis system (Promega, Madison, WI, USA). The activation degree of the target reporter gene was compared based on the ratio of measured relative luciferase (RLU) activity of firefly luciferase to that of Renilla luciferase. The predicted binding site fragments and mutated fragments of IGF2BP2 mRNA and miR-200b were inserted into the luciferase reporter vector as reporter plasmids, designated as IGF2BP2-WT and IGF2BP2-MUT. The IGF2BP2 mRNA luciferase reporter plasmid was co-transfected with NC or miR-200b mimic, respectively, to test whether IGF2BP2 can bind to miR-200b. The rest steps were performed as mentioned above.

### Anti-m(6)A Immunoprecipitation (MeRIP)

Total RNA was isolated from ESCC cells using the Trizol method, and mRNA in the total RNA was isolated and purified using PolyATtract^®^ mRNA Isolation Systems (Cat. Number: A-Z5300, Aide Technology Co., Ltd., Beijing, China). N6-methyladenosine (m6A) antibody (ab151230, 1: 500, Abcam) or IgG (ab109489, 1: 100, Abcam) was added into the IP buffer (20 mM Tris pH 7.5, 140 mM NaCl, 1% NP-40, 2 mM EDTA), and incubated for 1h with protein A/G magnetic beads for binding. The isolated and purified mRNA and magnetic beads-antibody complex was added into the IP buffer containing ribonuclease inhibitor and protease inhibitor and incubated overnight at 4°C. RNA was eluted with elution buffer, extracted, and purified by phenol-chloroform, and TK1 was analyzed by RT-qPCR.

### Photoactivatable-Ribonucleoside-Enhanced Crosslinking and Immunoprecipitation (PAR-CLIP)

ESCC cells were incubated with 200 mM 4-thiopyridine (4SU) (Sigma-Aldrich) for 14 h, and crosslinked under 0.4 J/cm^2^ irradiation at 365 nm. After lysis, immunoprecipitation was performed using IGF2BP2 antibodies (5 and 3 mg, respectively) at 4°C, and the precipitated RNA was labeled with [g-32-P]-ATP and observed by autoradiography. In PAR-fragment RT-qPCR analysis, proteinase K digestion was performed to remove proteins, and RT-qPCR was adopted to detect the expression of TK1 in extracted RNA.

### Tumor Xenografts

A stably transfected cell line was constructed for subcutaneous tumor formation in nude mice. In brief, ESCC cells were resuspended with serum-free RPMI 1640 medium (Gibco, Carlsbad, CA USA) to prepare 1 × 10^6^ cells/200 μL cell suspension for later use. Thirty-six BALB/c nude mice were randomly divided into three groups (n = 12/group). After the nude mice were anesthetized with diethyl ether, they were routinely sterilized, and 200 μL of cell suspension (1 × 10^6^ cells/200 μL) was injected subcutaneously into the right hind leg of the nude mice in each group (oe-NC + sh-NC group, oe-CCAT2 + sh-NC group, and oe-CCAT2 + sh-TK1 group). All mice were bred in the same environment. The mice and tumor inoculation sites were observed every day, and the volume of the transplanted tumor was measured and recorded every seven days, where tumor volume = (a * b^2^)/2 (a is the longest diameter and b the shortest diameter of the tumor). After four weeks, nude mice were euthanized by cervical dislocation, and the tumors were dissected, photographed, weighed, and measured.

### Statistical Analysis

All experimental data were analyzed using the SPSS 21.0 software (IBM Corp. Armonk, NY, USA). Measurement data were summarized as mean ± standard deviation from at least three independent experiments. Data between cancer tissues and normal adjacent tissues were compared by paired *t*-test. Data between remaining two groups were compared by unpaired *t*-test. Comparison among multiple groups was performed by one-way analysis of variance (ANOVA) with Tukey’s *post hoc* test. Comparison among groups at different time points was performed using repeated measures ANOVA with Bonferroni’s *post hoc* test. The Kaplan-Meier method was used to calculate the survival rate of patients, and Log-rank test was employed for single factor analysis. Pearson’s correlation analysis was adopted to analyze the relationship between the two indicators. *p* < 0.05 indicated that the difference was statistically significant, and ns indicates lack of statistical significance of a result.

## Results

### CCAT2 Is Highly Expressed in ESCC Cells and Tissues and Is Closely Related to Poor Prognosis of ESCC Patients

**Figure 1:**
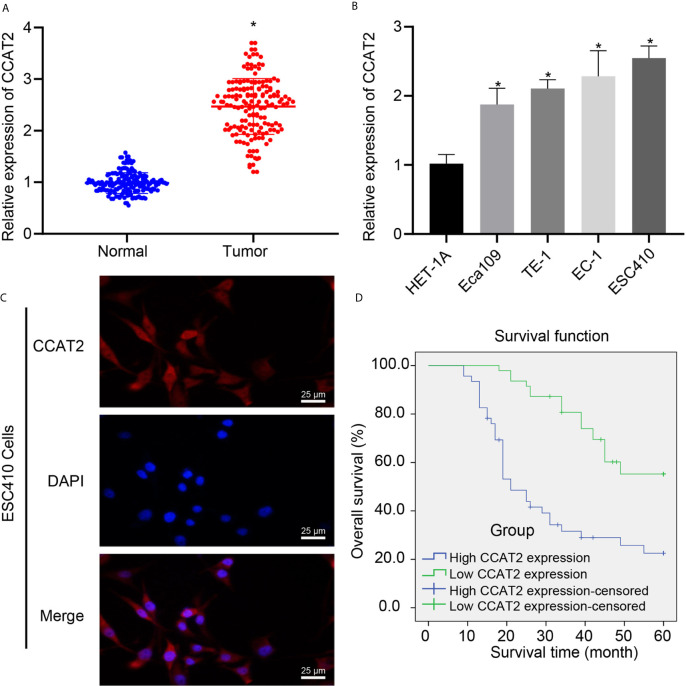
The expression of CCAT2 was upregulated in ESCC tissues and cells, and this upregulation indicated poor prognosis of ESCC patients. **(A)** RT-qPCR detection of CCAT2 expression in ESCC tissues and normal adjacent tissues. **(B)** RT-qPCR detection of CCAT2 expression in HET-1A human normal esophageal epithelial cells and four ESCC cell lines Eca109, TE-1, EC-1, and ESC410. **(C)** FISH analysis of subcellular localization of CCAT2. **(D)** Kaplan-Meier survival analysis (log-rank test) based on CCAT2 expression (n = 93). *p < 0.05 *vs.* normal adjacent tissues or HET-1A cell line. Data were shown as mean ± standard deviation of three technical replicates. Data between cancer tissues and normal adjacent tissues were compared by paired *t*-test. Data among multiple groups were compared by one-way ANOVA with Tukey’s *post hoc* test. Kaplan-Meier was adopted to calculate the survival rate of patients, and Log-rank test was used for univariate analysis.

CCAT2 has been proposed to be associated with poor prognosis of ESCC (7, 13), but its downstream regulatory mechanism had remained unknown. We utilized RT-qPCR to analyze the expression of CCAT2 in ESCC tissues and normal adjacent tissues, which showed that CCAT2 was highly expressed in ESCC tissues (**Figure 1A**). Similarly, we used RT-qPCR to examine CCAT2 expression in normal esophageal epithelial cell line HET-1A and 4 ESCC cell lines Eca109, TE-1, EC-1, and ESC410. The expression of CCAT2 was higher in the four ESCC cell lines than in HET-1A, with ESC410 exhibiting the very highest expression of CCAT2 (**Figure 1B**), which was accordingly used for subsequent experiments. The results of FISH revealed that CCAT2 was localized in both the cytoplasm and the nucleus (**Figure 1C**). The 93 patients with ESCC were divided into the high (expression > median value, n = 46) and low expression (expression ≤ median value, n = 47) groups according to the median value of the CCAT2 expression (**Supplementary Figure 1A**). The results showed that the overall survival of patients in the high expression group was lower than that in the low expression group (log rank = 20.055, *p* < 0.001) (**Figure 1D**), indicating that CCAT2 has a positive correlation with the occurrence and progression of ESCC. Taken together, CCAT2 is highly expressed in ESCC tissues and cells, and was strongly predictive of poor prognosis of ESCC patients.

### CCAT2 Promotes the Proliferation, Migration and Invasion of ESCC Cells and Inhibits Their Apoptosis *In Vitro*

**Figure 2:**
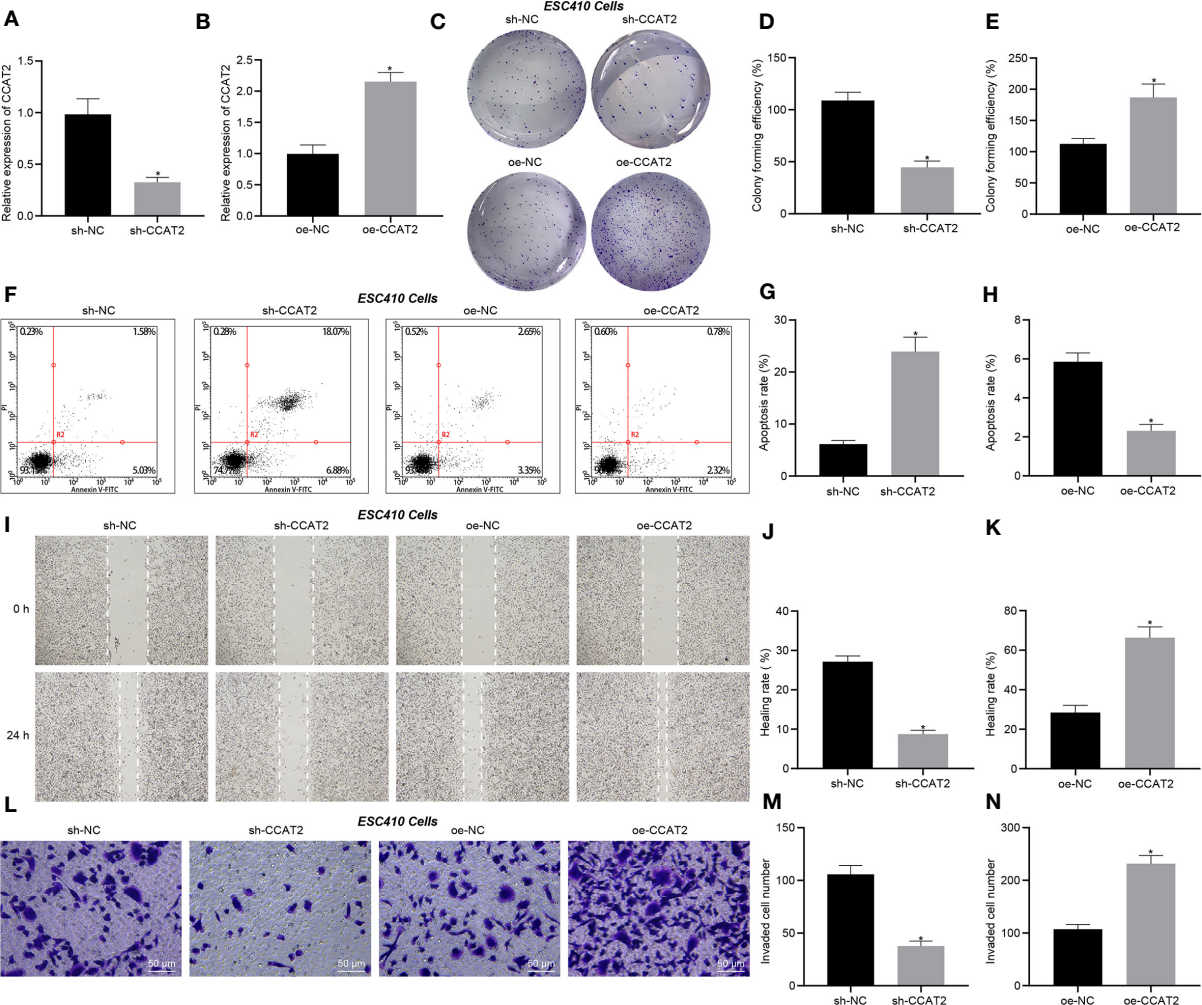
CCAT2 augments the proliferation, migration and invasion of ESCC cells and impedes their apoptosis *in vitro*. **(A)** RT-qPCR detection of CCAT2 expression in ESC410 cells treated with sh-NC or sh-CCAT2. **(B)** RT-qPCR detection of CCAT2 expression in ESC410 cells treated with oe-NC or oe-CCAT2. **(C–E)** Colony formation assay detection of ESC410 cell proliferation after overexpressing or silencing CCAT2. **(F–H)** Flow cytometric analysis of ESC410 cell apoptosis after overexpressing or silencing CCAT2. **(I–K)** Scratch assay detection of ESC410 cell migration after overexpressing or silencing CCAT2. **(L–N)** Transwell assay detection of ESC410 cell invasion after overexpressing or silencing CCAT2, scale bar = 50 μm. *p < 0.05 *vs.* oe-NC-treated cells or sh-NC-treated cells. Data were shown as mean ± standard deviation of three technical replicates. Data between two groups were compared by unpaired *t*-test.

Transfection efficiency of CCAT2 in ESC410 cells was validated using RT-qPCR (**Figures 2A, B**). Compared with the cells injected with si-NC, inhibition of CCAT2 expression in ESC410 cells impaired cell proliferation ability (**Figures 2C, D**), increased cell apoptosis (**Figures 2F, G**), and inhibited the migration and invasion ability of the ESC410 cells (**Figures 2I, J, L, M**). However, overexpression of CCAT2 in ESC410 cells presented totally opposite effects (**Figures 2C, E, F, H, I, K, L, N**). In summary, CCAT2 could stimulate the proliferation, migration and invasion of ESCC cells and inhibits their apoptosis *in vitro*.

### CCAT2 Promotes the Proliferation, Migration, and Invasion of ESCC Cells by Binding to miR-200b

**Figure 3:**
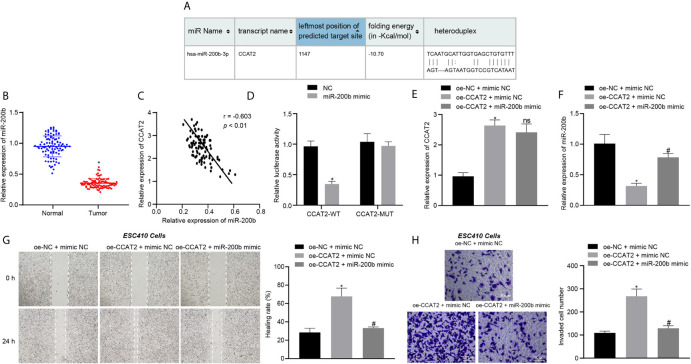
CCAT2 binds to miR-200b and reduces its expression, thus triggering the proliferation, migration and invasion of ESCC cells. **(A)** The binding site between CCAT2 and miR-200b predicted by the RNA22 database. **(B)** RT-qPCR detection of miR-200b expression in 93 cases of ESCC tissues and normal adjacent tissues. **(C)** Correlation analysis of CCAT2 expression and miR-200b expression in 93 cases of ESCC tissues by Pearson’s correlation analysis. **(D)** Binding of CCAT2 to miR-200b confirmed by dual luciferase report assay. **(E)** RT-qPCR detection of the expression of CCAT2 in ESC410 cells after overexpression of CCAT2 and miR-200b. **(F)** RT-qPCR detection of the expression of miR-200b in ESC410 cells after overexpression of CCAT2 and miR-200b. **(G)** Scratch assay detection of ESC410 cell migration after overexpression of CCAT2 and miR-200b. **(H)** Transwell assay detection of ESC410 cell invasion after overexpression of CCAT2 and miR-200b. Scale bar = 50 μm. *p < 0.05 *vs.* oe-NC + mimic NC-treated cells. ^#^ *p* < 0.05 *vs.* oe-CCAT2 + mimic NC-treated cells. Data were shown as mean ± standard deviation of three technical replicates. Data between cancer tissues and normal adjacent tissues were compared by paired *t*-test. Data between remaining two groups were compared by unpaired *t*-test. Data among multiple groups were compared by one-way ANOVA with Tukey’s *post hoc* test.

We then aimed to explore the mechanism of CCAT2 in ESCC cells. The RNA22 database predicted the presence of binding sites between CCAT2 and miR-200b (**Figure 3A**). The expression of miR-200b in 93 ESCC tissues and normal adjacent tissues was analyzed using RT-qPCR. The results showed that the expression of miR-200b was reduced in ESCC tissues (**Figure 3B**), while CCAT2 expression was elevated in the tumor samples (**Figure 3C**). These results suggested that CCAT2 may bind to miR-200b in ESCC. Furthermore, the results of dual luciferase reporter assay showed that the luciferase activity of cells co-transfected with miR-200b mimic and CCAT2-WT plasmid was lower when compared with cells co-transfected with NC and CCAT2-WT plasmid (**Figure 3D**), suggesting that CCAT2 can bind to miR-200b. In addition, compared with the oe-NC + mimic NC-treated ESC410 cells, the expression of CCAT2 was increased in the oe-CCAT2 + mimic NC-treated ESC410 cells (**Figure 3E**), while that of miR-200b was reduced (**Figure 3F**), and the migration and invasion ability of ESC410 cells was enhanced (**Figures 3G, H**). However, after we further overexpressed miR-200b in cells (treated with oe-CCAT2 + miR-200b mimic), there was no significant change in CCAT2 expression, while miR-200b expression was increased (**Figure 3F**), and reduced migration and invasion ability of ESC410 cells was observed (**Figures 3G, H**). Taken together, CCAT2 can target miR-200b to reduce its expression in ESCC, and consequently induces the proliferation, migration and invasion of ESCC cells.

### CCAT2 Upregulates IGF2BP2 Expression by Binding to miR-200b to Promote the Migration and Invasion of ESCC Cells

**Figure 4:**
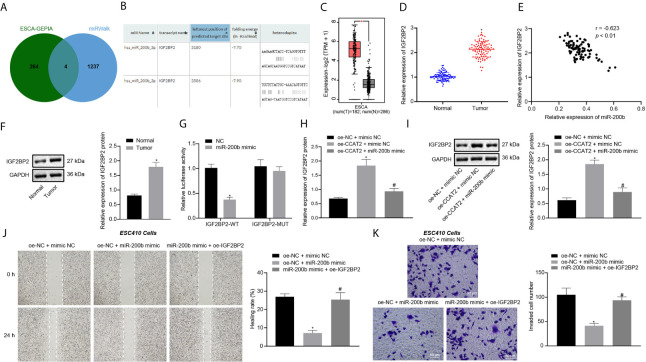
CCAT2 upregulates the expression of IGF2BP2 by adsorbing miR-200b to promote the migration and invasion of ESCC cells. **(A)** Venn diagram of differentially expressed genes from GEPIA analysis and downstream genes of miR-200b obtained by miRWalk, and the intersection genes are CXCL8, LAMC2, IGF2BP2, and EPHB2. **(B)** The binding site between miR-200b and IGF2BP2 predicted by the RNA22 database. **(C)** The box diagram of IGF2BP2 expression through GEPIA analysis; the left red box indicates the expression in ESCC samples, and the right gray box indicates the expression in normal samples. **(D)** RT-qPCR detection of mRNA expression of IGF2BP2 in 93 cases of ESCC tissues and normal adjacent tissues. **(E)** Correlation analysis of IGF2BP2 mRNA expression and miR-200b expression in 93 cases of ESCC tissues by Pearson’s correlation analysis. **(F)** Immunoblotting analysis of IGF2BP2 protein in 93 cases of ESCC tissues and normal adjacent tissues. **(G)** Binding of miR-200b to IGF2BP2 confirmed by dual luciferase report assay. **(H, I)** RT-qPCR and immunoblotting detection of the mRNA and protein expression of IGF2BP2 in ESC410 cells after overexpression of CCAT2 and miR-200b. **(J)** Scratch assay detection of ESC410 cell migration ability in each group. **(K)** Transwell assay detection of ESC410 cell invasion ability in each group, scale bar = 50 μm. *p < 0.05 *vs.* normal adjacent tissues, HET-1A cell line, oe-NC-treated cells, sh-NC-treated cells, or oe-NC + mimic NC-treated cells. ^#^ *p* < 0.05 *vs.* oe-CCAT2 + mimic NC-treated cells or oe-NC + miR-200b mimic-treated cells. Data were shown as mean ± standard deviation of three technical replicates. Data between cancer tissues and normal adjacent tissues were compared by paired *t*-test. Data between remaining two groups were compared by unpaired *t*-test. Data among multiple groups were compared by one-way ANOVA with Tukey’s *post hoc* test.

GEPIA analysis on the ESCC data in TCGA database revealed 268 differentially expressed genes (**Supplementary Figure 1B**). A total of 1241 downstream genes of miR-200b were predicted by the miRWalk database. The intersection analysis of the predicted results by the two databases suggested four differentially expressed downstream genes: CXCL8, LAMC2, IGF2BP2, and EPHB2 (**Figure 4A**). A previous study has shown that IGF2BP2 is related to the development of ESCC (10). Moreover, we predicted that miR-200b could target and bind with IGF2BP2 through RNA22 (**Figure 4B**). IGF2BP2 was also highly expressed in ESCC samples according to GEPIA analysis (**Figure 4C**). To validate this finding, RT-qPCR and immunoblotting were untaken, which displayed that the expression of IGF2BP2 was increased in ESCC tissues (**Figures 4D, F**), where it showed an inverse correlation with miR-200b expression (**Figure 4E**). The above results indicated that miR-200b may target IGF2BP2 in ESCC. Additionally, the results of dual luciferase reporter assay showed that the luciferase activity of cells co-transfected with miR-200b mimic and IGF2BP2-WT plasmid was lower when compared with cells co-transfected with NC and IGF2BP2-WT plasmid (**Figure 4G**), thus confirming the binding of miR-200b to IGF2BP2.

RT-qPCR and immunoblotting results revealed that, compared with the oe-NC + mimic NC-treated cells, the expression of IGF2BP2 was increased in the oe-CCAT2 + mimic NC-treated cells (**Figures 4H, I**). However, this trend was reversed by further overexpression of miR-200b in ESC410 cells (**Figures 4H, I**), demonstrating that CCAT2 upregulated IGF2BP2 expression by adsorbing miR-200b.

The results of scratch and Transwell assays presented that, compared with the oe-NC + mimic NC-treated cells, the invasion and migration ability of oe-NC + miR-200b mimic-treated ESC410 cells were reduced (**Figures 4J, K**). However, when compared with overexpression of miR-200b alone, simultaneous overexpression of miR-200b and IGF2BP2 promoted the invasion and migration ability of ESC410 cells (**Figures 4J, K**). To summarize, CCAT2 upregulated the expression of IGF2BP2 by adsorbing miR-200b to promote ESCC cell migration and invasion.

### IGF2BP2 Maintains TK1 mRNA Stability and Promotes Its Expression by Recognizing the m6A Modification of TK1 mRNA

**Figure 5:**
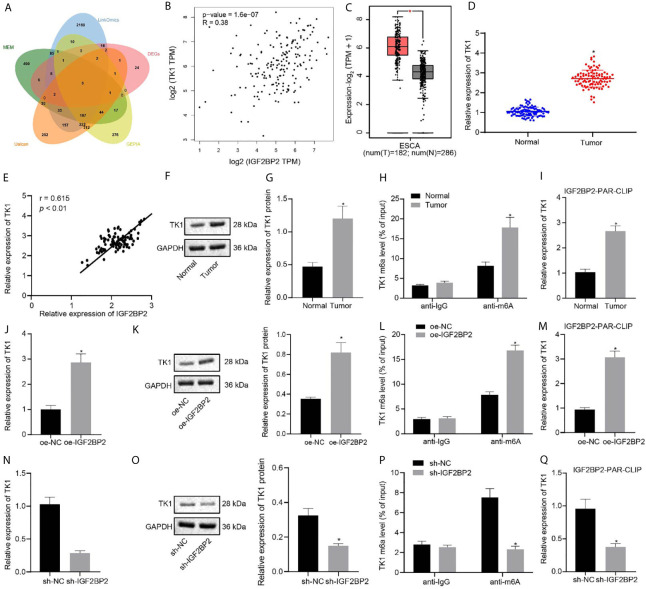
IGF2BP2 maintains TK1 mRNA stability to promote TK1 expression by recognizing the m6A modification of TK1 mRNA. **(A)** Venn diagram of differentially expressed genes and related genes of IGF2BP2 obtained from Ualcan, LinkedOmics, GEPIA, and MEM, and the six intersection genes are FSCN1, ECT2, TPX2, MCM2, FANCI, and TK1. **(B)** Correlation map of IGF2BP2 expression and TK1 expression obtained by GEPIA analysis (*p* = 1.6e-07). **(C)** The box diagram of TK1 expression through GEPIA analysis; the left red box indicates the expression in ESCC samples, and the right gray box indicates the expression in normal samples. **(D)** RT-qPCR detection of mRNA expression of TK1 in 93 cases of ESCC tissues and normal adjacent tissues. **(E)** Correlation analysis of TK1 mRNA expression and IGF2BP2 mRNA expression in 93 cases of ESCC tissues by Pearson’s correlation analysis. **(F, G)**. Immunoblotting detection of TK1 protein in 93 cases of ESCC tissues and normal adjacent tissues. **(H)** Me-RIP detection of the m6A modification level of TK1 in ESCC tissues and normal adjacent tissues. **(I)** PAR-CLIP detection of the binding between IGF2BP2 and TK1 mRNA in ESCC tissues and normal adjacent tissues. **(J, K)**. RT-qPCR and immunoblotting detection of the mRNA and protein expressions of TK1 in oe-NC-treated and oe-IGF2BP2-treated cells. **(L)** Me-RIP detection of the m6A modification level of TK1 in oe-NC-treated and oe-IGF2BP2-treated cells. **(M)** PAR-CLIP detection of the binding between IGF2BP2 and TK1 mRNA in ESC410 cells of each group. **(N, O)** RT-qPCR and immunoblotting detection of the mRNA and protein expressions of TK1 in sh-NC-treated and sh-IGF2BP2-treated cells. **(P)** Me-RIP detection of the m6A modification level of TK1 in sh-NC-treated and sh-IGF2BP2-treated cells. **(Q)** PAR-CLIP detection of the binding between IGF2BP2 and TK1 mRNA in sh-NC-treated and sh-IGF2BP2-treated cells. *p < 0.05 *vs.* normal adjacent tissues, oe-NC-treated cells, or sh-NC-treated cells. Data were shown as mean ± standard deviation of three technical replicates. Data between cancer tissues and normal adjacent tissues were compared by paired *t*-test. Data between the remaining two groups were compared by unpaired *t*-test.

Through GEPIA differential analysis of ESCC data in TCGA, 4,049 differentially expressed genes were obtained (**Supplementary Figure 1C**). According to datasets GSE20347, GSE29001, GSE38129, GSE45168, GSE45350, and GSE45670, we obtained 993, 1860, 725, 1973, 2768, and 2594 differentially expressed genes, respectively, which yielded 70 intersected and differentially expressed genes. Besides, 1172, 2821, 1000, and 831 related genes of IGF2BP2 were acquired by Ualcan, LinkedOmics, GEPIA, and MEM, respectively. The intersection of above differentially expressed genes and related genes yielded six key genes: FSCN1, ECT2, TPX2, MCM2, FANCI, and TK1 (**Figure 5A**). Previous studies have shown that TK1 has a close relationship with ESCC (11, 14). Further analysis by GEPIA indicated that IGF2BP2 and TK1 had a significant correlation of expression (**Figure 5B**), and that TK1 expression was elevated in ESCC samples (**Figure 5C**). Given that IGF2BP2 was reported as an m6A modification-related gene (12), we speculated that IGF2BP2 might tune TK1 expression through m6A modification. The results of RT-qPCR and immunoblotting displayed that TK1 expression was increased in ESCC tissues (**Figures 5D, F, G**), where TK1 was positively correlated to the mRNA expression of IGF2BP2 (**Figure 5E**). The level of m6A modification of TK1 in ESCC tissues and adjacent normal tissues was detected by Me-RIP experiments, which demonstrated that the level of m6A modification of TK1 in ESCC tissues was indeed increased (**Figure 5H**). The results of PAR-CLIP test exhibited that the binding between IGF2BP2 and TK1 mRNA was enhanced in ESCC tissues (**Figure 5I**). The ESC410 cells were transfected with oe-NC, oe-IGF2BP2, sh-NC, or sh-IGF2BP2. As revealed by RT-qPCR, immunoblotting and Me-RIP, compared with the oe-NC-treated cells, the mRNA and protein expressions of TK1 and the m6A modification level of TK1 in the oe-IGF2BP2-treated cells were increased (**Figures 5J–L**), and the binding between IGF2BP2 and TK1 mRNA was also increased (**Figure 5M**). However, silencing IGF2BP2 presented totally opposite effects (**Figures 5N–Q**). Together, IGF2BP2 enhanced the stability of TK1 mRNA to promote its expression by recognizing the m6A modification of TK1 mRNA.

### CCAT2 Promotes the Migration and Invasion of ESCC Cells by Upregulating TK1

**Figure 6:**
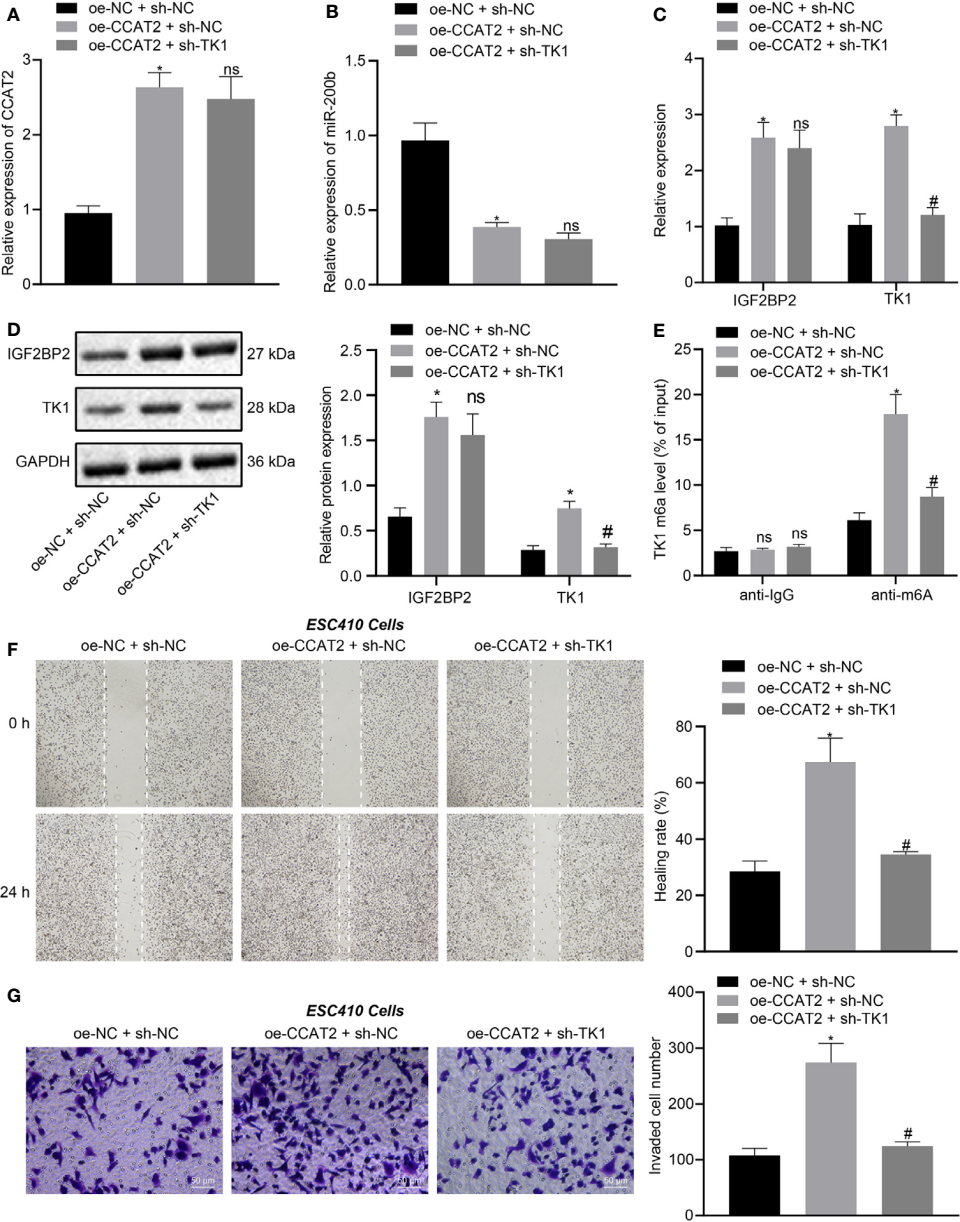
CCAT2 upregulates TK1 to promote the migration and invasion of ESCC cells *in vitro*. **(A, B)**. RT-qPCR detection of the expression of CCAT2 and miR-200b in oe-NC + sh-NC-, oe-CCAT2 + sh-NC-, and oe-CCAT2 + sh-TK1-treated ESC410 cells. **(C, D)**. RT-qPCR and immunoblotting detection of the mRNA and protein expression of IGF2BP2 and TK1 in ESC410 cells of each group. **(E)** Me-RIP detection of m6A modification level of TK1 in ESC410 cells of each group. **(F)** Scratch assay detection of the migration ability of ESC410 cells in each group. **(G)** Transwell assay detection of the invasion ability of ESC410 cells in each group, scale bar = 50 μm. *p < 0.05 *vs.* oe-NC + sh-NC-treated cells; ^#^ *p* < 0.05 *vs.* oe-CCAT2 + sh-NC-treated cells; ns indicates no significant difference. Data were shown as mean ± standard deviation of three technical replicates. Data between two groups were compared by unpaired *t*-test. Data among multiple groups were compared by one-way ANOVA with Tukey’s *post hoc* test.

ESC410 cells were transfected with oe-NC + sh-NC, oe-CCAT2 + sh-NC, and oe-CCAT2 + sh-TK1. RT-qPCR was then used to detect the expression of CCAT2 and miR-200b in cells of each group. Compared with untreated cells, the expression of CCAT2 was increased in the oe-CCAT2 + sh-NC-treated cells (**Figure 6A**), miR-200b expression was decreased (**Figure 6B**), the mRNA and protein expression of IGF2BP2 and TK1 were increased (**Figures 6C, D**), the m6A modification level of TK1 was increased (**Figure 6E**), and the invasion and migration ability of ESC410 cells was increased, as reflected by scratch and Transwell assays (**Figures 6F, G**). When compared with oe-CCAT2 + sh-NC-treated cells, there was no significant change in the expression of CCAT2, miR-200b, and IGF2BP2 in the oe-CCAT2 + sh-TK1-treated cells (**Figures 6A–D**). However, the expression of TK1 and m6A modification level of TK1 mRNA were reduced (**Figures 6C–E**), and the invasion and migration ability of ESC410 cells was reduced in response to treatment with oe-CCAT2 + sh-TK1 (**Figures 6F, G**). Taken together, CCAT2 upregulated IGF2BP2 expression by adsorbing miR-200b to enhance the expression of TK1, which promoted the migration and invasion of ESCC cells *in vitro*.

### CCAT2 Promoted the Tumorigenesis of ESCC Cells in Nude Mice by Regulating TK1 Expression

**Figure 7:**
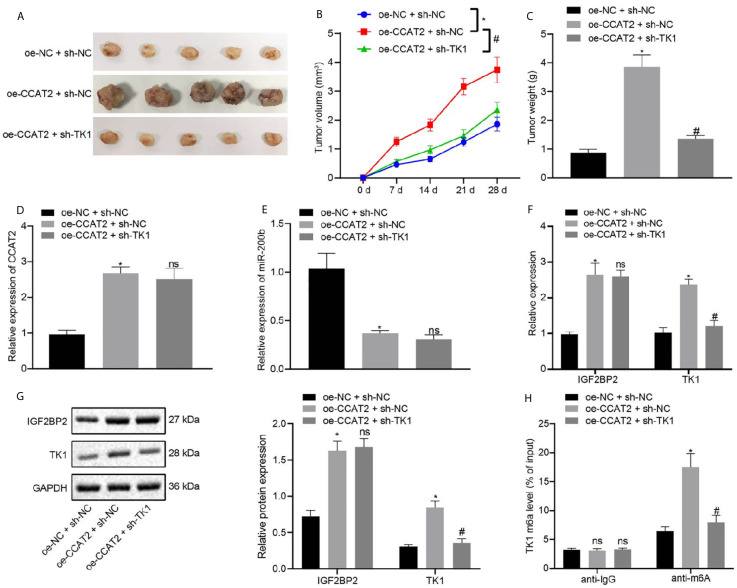
CCAT2 upregulates the expression of TK1 to promote the tumorigenesis of ESCC cells in nude mice. **(A)** Representative images showing xenografts in nude mice of each group. **(B)** Changes in the volume of formed tumors within four weeks of implanting ESC410 cells transfected with different plasmids in each group. **(C)** The weight of formed tumors within four weeks of implanting ESC410 cells in each group. **(D, E)** RT-qPCR detection of the expression of CCAT2 and miR-200b in cancer tissues of oe-NC + sh-NC-treated mice, oe-CCAT2 + sh-NC-treated mice, and oe-CCAT2 + sh-TK1-treated mice. **(F, G)** RT-qPCR and immunoblotting detection of mRNA and protein expressions of IGF2BP2 and TK1 in cancer tissues of each group. **(H)** Me-RIP detection of the m6A modification level of TK1 in cancer tissues of each group. *p < 0.05 *vs.* oe-NC + sh-NC-treated mice. ^#^ *p* < 0.05 *vs.* oe-CCAT2 + sh-NC-treated mice. Data were shown as mean ± standard deviation of three technical replicates. Data among multiple groups were compared by one-way ANOVA with Tukey’s *post hoc* test. Comparison among groups at different time points was performed using repeated measures ANOVA with Bonferroni’s *post hoc* test. ns means no significant difference.

In order to elucidate the effects of CCAT2 and TK1 on the growth of transplanted ESCC tumors *in vivo*, we conducted subcutaneous tumor xenografts experiments in nude mice. The mice were subcutaneously injected with ESC410 cells transfected with oe-NC + sh-NC, oe-CCAT2 + sh-NC, or oe-CCAT2 + sh-TK1, and their tumor volume measured every week. Compared with the sh-NC + oe-NC-treated mice, the tumor growth of the oe-CCAT2 + sh-NC-treated mice was increased, (**Figures 7A–C**), CCAT2 expression was increased (**Figure 7D**), miR-200b expression was decreased (**Figure 7E**), mRNA and protein expression of IGF2BP2 and TK1 were increased (**Figures 7F, G**), and the m6A modification level of TK1 was also increased (**Figure 7H**). When compared with oe-CCAT2 + sh-NC-treated mice, tumorigenic ability was reduced and there was no significant change in the expression of CCAT2, miR-200b, and IGF2BP2 in the oe-CCAT2 + sh-TK1-treated mice (**Figures 7D–G**); however, the expression of TK1 and m6A modification level of TK1 mRNA were reduced (**Figures 7E, G, H**). Taken together, CCAT2 promoted the tumorigenesis of ESCC cells in nude mice by upregulating the expression of TK1.

## Discussion

ESCC, which is the most common subtype of esophageal cancer, and an increasing incidence worldwide and is associated with poor prognosis (15). The etiology of ESCC, in addition to the genetic components, is attributed largely to modifiable environmental components, such as smoking, alcohol intake, and habitual intake of very hot drinks (16). To improve the overall survival rate of patients, besides providing more accessible and affordable endoscopy for early diagnosis (17), developing novel and effective therapeutic treatments is of great importance, which heavily relies on obtaining a better understanding of the regulatory pathways in ESCC. In our study, we found that CCAT2 binds to miR-200b to suspend its inhibitory effects on IGF2BP2 expression, that resulting in elevated TK1 expression, which promoted the development of ESCC.

It has been reported that the expression of CCAT2 is upregulated in ESCC tissues (18). Consistent with this notion, our RT-qPCR data also confirmed that CCAT2 was highly expressed in ESCC tissues and cell lines. Moreover, we found that CCAT2 expression was positively correlated with the occurrence and deterioration of ESCC, a finding also supported by previous literature (7). To investigate the specific role of CCAT2 in ESCC, we suppressed the expression of CCAT2 in an ESCC cell line, and discovered that this inhibition of CCAT2 expression impaired cell proliferation ability, while increased cell apoptosis, and inhibited the migration and invasion ability of ESCC cells. Consistent with that finding, CCAT2 expression was overexpressed in triple-negative breast cancer, and its oncogenic function was validated both *in vitro* and *in vivo* (19). In the context of esophageal carcinoma, CCAT2 also serves as an oncogene, which promotes radiotherapy resistance (20).

It has been revealed that in osteosarcoma, CCAT2 serves as an oncogene to regulate miR-200b/VEGF axis to enhance cell progression and cell mobility (9). More importantly, miR-200b was reported to suppress cell growth and invasion in ESCC (21, 22). To further investigate such a developing pathway, we profiled the downstream genes of miR-200b through miRWalk, of which IGF2BP2 stood out as a potential target involved in the regulating pathway of ESCC. The underlying rationale here is that IGF2BP2 expression was reported to be upregulated in ESCC (10), which was also verified by the present GEPIA analysis, RT-qPCR, and immunoblotting results. The impact of IGF2BP2 expression on the biological characteristics of ESCC cells was recently reported (23), and our present results of scratch and Transwell assays concur with that result, in displaying that CCAT2 upregulated the expression of IGF2BP2 by adsorbing miR-200b in ESCC cells to promote their migration and invasion capacity.

Furthermore, in our subsequent study, we found that IGF2BP2 and TK1 have significantly correlated expression in tumor cells, and our bioinformatics analysis data unraveled that TH1 expression was abnormally elevated in ESCC. Most importantly, serum TK1 levels were reported to be highly associated with clinicopathological features in patients with ESCC (24). Besides, IGF2BP2 has been widely reported to be involved in the dynamic regulation of m6A modification of its target gene (25), thus making it highly plausible that IGF2BP2 may tune TK1 expression through regulating the m6A modification of TK1 mRNA. Me-RIP experimental results explained that the increase of m6A modification of TK1 in ESCC tissues may account for such expressional upregulation (26), and the PAR-CLIP test revealed that IGF2BP2 recognized m6A modification of TK1 mRNA to maintain its stability. Finally, we validated such a regulatory pathway both in a cellular model and a nude mouse ESCC model, and our data furthermore unraveled that CCAT2 promoted the migration and invasion of ESCC cells *in vitro*, and tumorigenesis *in vivo* by upregulating TK1 expression.

**Figure 8:**
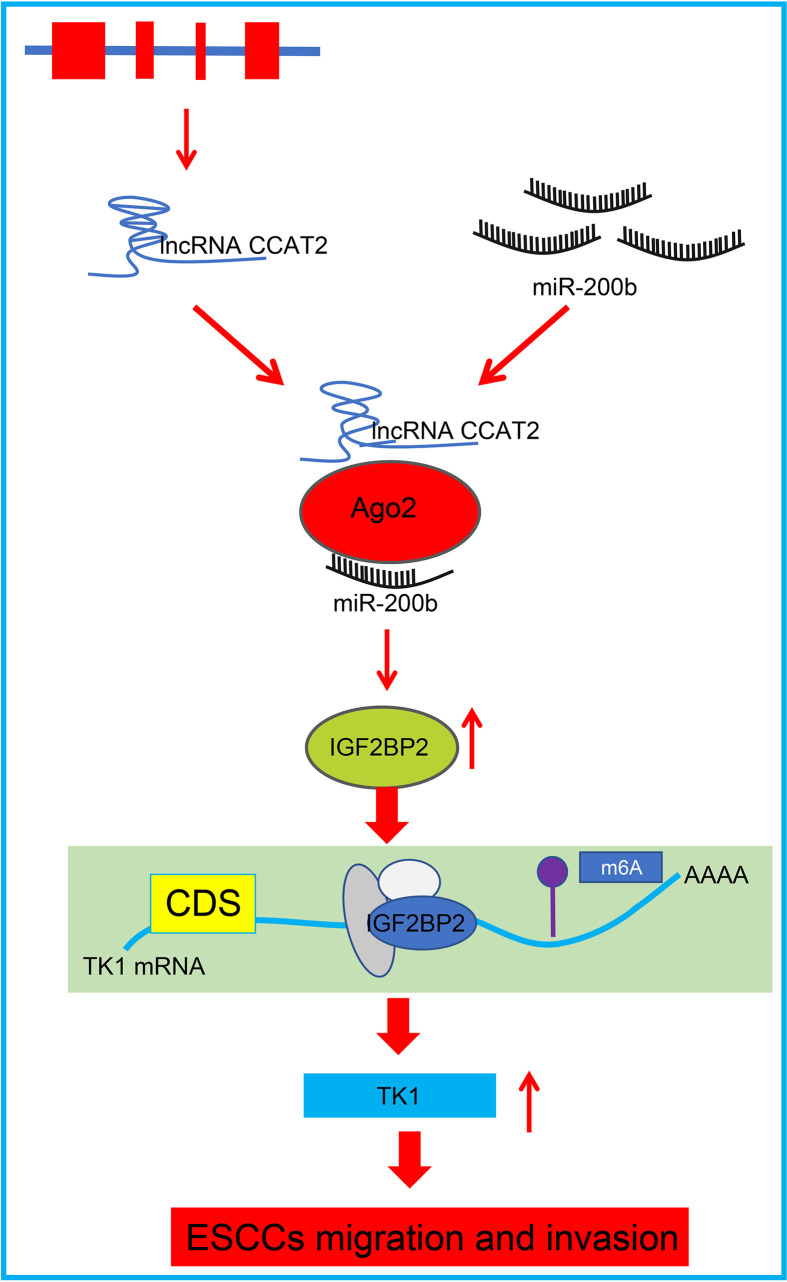
Schematic diagram of the mechanism by which CCAT2 affects development and progression of ESCC. CCAT2 binds with miR-200b to reduce its expression, and consequently enhances the expression of the miR-200b target IGF2BP2, which leads to upregulated TK1 expression and promotion of migration and invasion of ESCC cells *in vitro*.

In summary, our data present that CCAT2 expression was upregulated in ESCC cells and patient tissues. CCAT2 binds with miR-200b to enhance IGF2BP2 expression, which in turn upregulated TK1 expression to promote migration and invasion of ESCC cells *in vitro*, while accelerating tumorigenesis *in vivo*; this phenotype could be reversed by overexpressing miR-200b (**Figure 8**). This study this presents CCAT2 as a new therapeutic target for the treatment of esophageal cancer. However, further preclinical and clinical are required to determine the translational value of this mechanism in clinical application.

## Data Availability Statement

The original contributions presented in the study are included in the article/**Supplementary Material**. Further inquiries can be directed to the corresponding author.

## Ethics Statement

The studies involving human participants were reviewed and approved by the Ethics Committee of Nantong Tumor Hospital. The patients/participants provided their written informed consent to participate in this study. The animal study was reviewed and approved by the Animal Experiment Ethics Committee of Nantong Tumor Hospital.

## Author Contributions

XW: methodology, data curation, formal analysis, investigation, and writing - original draft. YF: methodology, resources, investigation, validation, and writing - original draft. YL: conceptualization, visualization, resources, investigation, and validation. BS: conceptualization, visualization, and project administration. HL: writing - review and editing, and project administration. HM: writing - review and editing, and supervision. All authors contributed to the article and approved the submitted version.

## Conflict of Interest

The authors declare that the research was conducted in the absence of any commercial or financial relationships that could be construed as a potential conflict of interest.

## Publisher’s Note

All claims expressed in this article are solely those of the authors and do not necessarily represent those of their affiliated organizations, or those of the publisher, the editors and the reviewers. Any product that may be evaluated in this article, or claim that may be made by its manufacturer, is not guaranteed or endorsed by the publisher.
