# Supplementary material for: Long Non-Coding RNA CCAT2 Promotes the Development of Esophageal Squamous Cell Carcinoma by Inhibiting miR-200b to Upregulate the IGF2BP2/TK1 Axis
Source: Front Oncol. 2021 Jul 27;11:680642. doi: 10.3389/fonc.2021.680642 (PMC8353391; doi:10.3389/fonc.2021.680642)
Supplement: Supplementary file 1 [file DataSheet_1.docx]

**
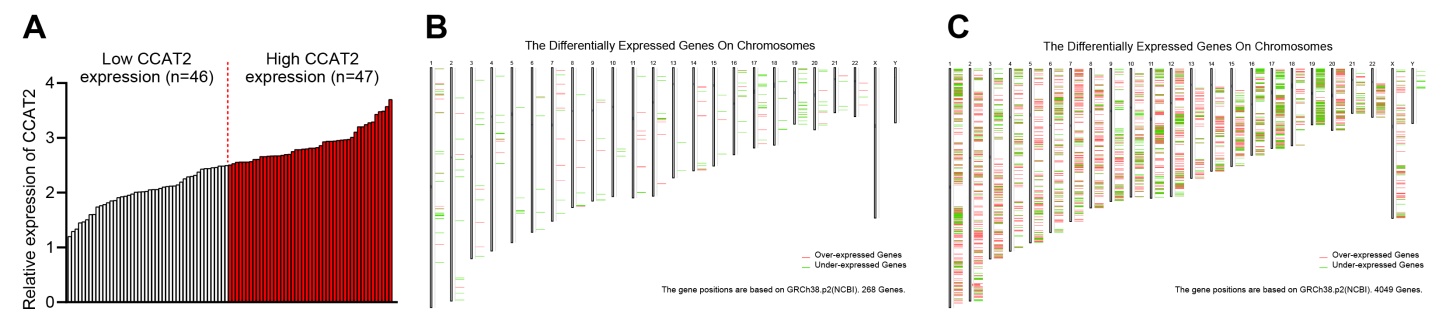
**

**SUPPLEMENTARY FIGURE 1.** CCAT2 expression classification and bioinformatics analysis of ESCC data. A. According to the median expression of CCAT2 in ESCC tissues, patients were divided into a high CCAT2 expression group and a low CCAT2 expression group (n = 93). B. The expression position of the differentially expressed genes on the chromosome obtained from the ESCC data in TCGA database analyzed by GEPIA as well as the corresponding heat map; red indicates upregulated genes and green indicates downregulated genes. C. The expression position of the differentially expressed genes on the chromosome obtained from the ESCC data in TCGA database analyzed by GEPIA as well as the corresponding heat map; red indicates upregulated genes and green indicates downregulated genes.
