# Supplementary material for: Long Non-Coding RNA CCAT2 Promotes the Development of Esophageal Squamous Cell Carcinoma by Inhibiting miR-200b to Upregulate the IGF2BP2/TK1 Axis
Source: Front Oncol. 2021 Jul 27;11:680642. doi: 10.3389/fonc.2021.680642 (PMC8353391; doi:10.3389/fonc.2021.680642)
Supplement: Supplementary file 2 [file Table_1.docx]

**SUPPLEMENTARY TABLE 1.** Information of the dataset retrieved from the GEO database

| GSE_ID | Platform | Normal sample | ESCA sample | Sample size |
| --- | --- | --- | --- | --- |
| GSE20347 | GPL571 | 17 | 17 | 34 |
| GSE29001 | GPL571 | 24 | 21 | 45 |
| GSE38129 | GPL571 | 30 | 30 | 60 |
| GSE45168 | GPL13497 | 5 | 5 | 10 |
| GSE45350 | GPL13607 | 4 | 4 | 8 |
| GSE45670 | GPL570 | 10 | 28 | 38 |

**SUPPLEMENTARY TABLE 2.** Primer sequences used for RT-qPCR

| Targets | Primer sequences (5’-3’) |
| --- | --- |
| CCAT2 | F: 5’-AGACAGTGCCAGCCAACC-3’ |
|  | R: 5’-TGCCAAACCCTTCCCTTA-3’ |
| miR-200b | F: 5’-TAATACTGCCTGGTAATGATGA-3’ |
|  | R: 5’-AACGCTTCACGAATTTGCGT-3’ |
| IGF2BP2 | F: 5’-GTTCCCGCATCATCACTCTTAT-3’ |
|  | R: 5’-GAATCTCGCCAGCTGTTTGA-3’ |
| TK1 | F: 5’-ACACATGACCGGAACACC-3’ |
|  | R: 5’-CAGAACTCCACGATGTCAGG-3’ |
| U6 | F: 5’-CTCGCTTCGGCAGCACA-3’ |
|  | R: 5’-AACGCTTCACGAATTTGCGT-3’ |
| GAPDH | F: 5’-AATGGACAACTGGTCGTGGAC-3’ |
|  | R: 5’-CCCTCCAGGGGATCTGTTTG-3’ |

Notes: RT-qPCR, reverse transcription quantitative polymerase chain reaction; F, forward; R, reverse; CCAT2, colon cancer-associated transcript 2 gene; miR-200b, microRNA-200b; IGF2BP2, insulin-like growth factor 2 mRNA-binding protein 2; TK1, thymidine kinase 1; U6, U6 small nuclear RNA; GAPDH, glyceraldehyde-3-phosphate dehydrogenase.
